# Supplementary material for: Concept and location neurons in the human brain provide the ‘what’ and ‘where’ in memory formation
Source: Nat Commun. 2024 Sep 10;15:7926. doi: 10.1038/s41467-024-52295-5 (PMC11387663; doi:10.1038/s41467-024-52295-5)
Supplement: Supplementary file 1 — Supplementary Information [file 41467_2024_52295_MOESM1_ESM.pdf]

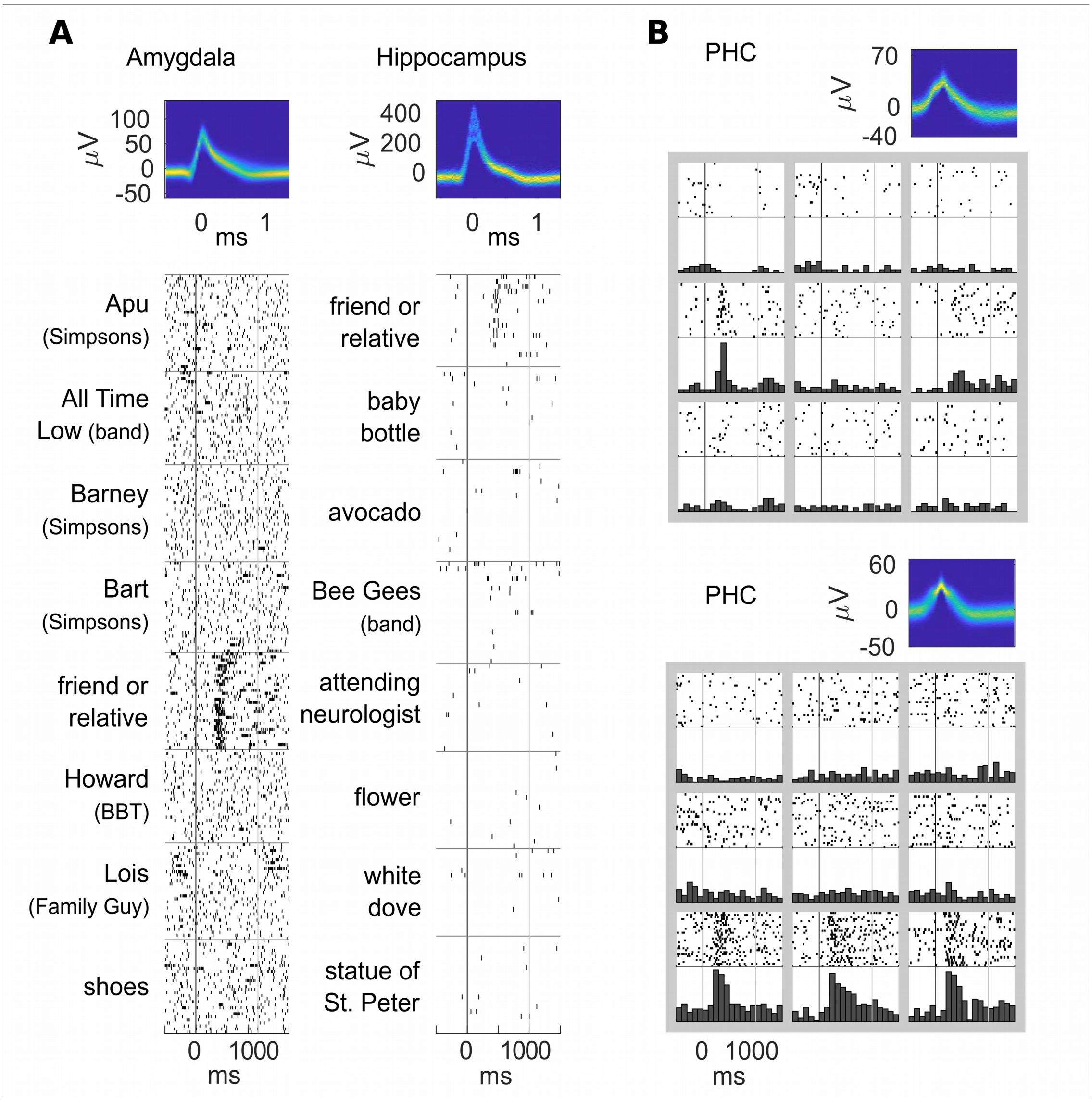

Figure S1. **Full raster plots.** (A) Responses of the two neurons shown in Fig. 2(A) to all presented items. (B) Responses of the two neurons shown in Fig 2(B) to all locations.
Source data are provided in a git repository (see Data Availablity).
Note that the original stimuli have been replaced by written labels to comply with publication requirements. This includes characters from TV shows, specifically The Simpsons (Apu, Barney, Bart), The Big Bang Theory (BBT; Howard), Family Guy (Lois), and pictures of CD covers by the bands ‘All Time Low’ and ‘Bee Gees’.

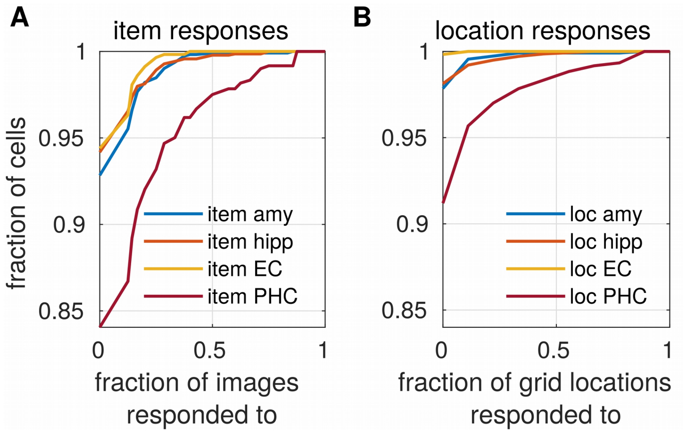

Figure S2. Selectivity of responses to items and spatial locations. Cumulative fractions of (A) response-eliciting items and (B) spatial locations (loc) for different MTL regions. Note that the x values in panel (A) are not evenly spaced due to different total numbers of images used per session (Methods).

amy, amygdala; hipp, hippocampus; EC, entorhinal cortex; PHC, parahippocampal cortex.

Source data are provided in a git repository (see Data Availablity).

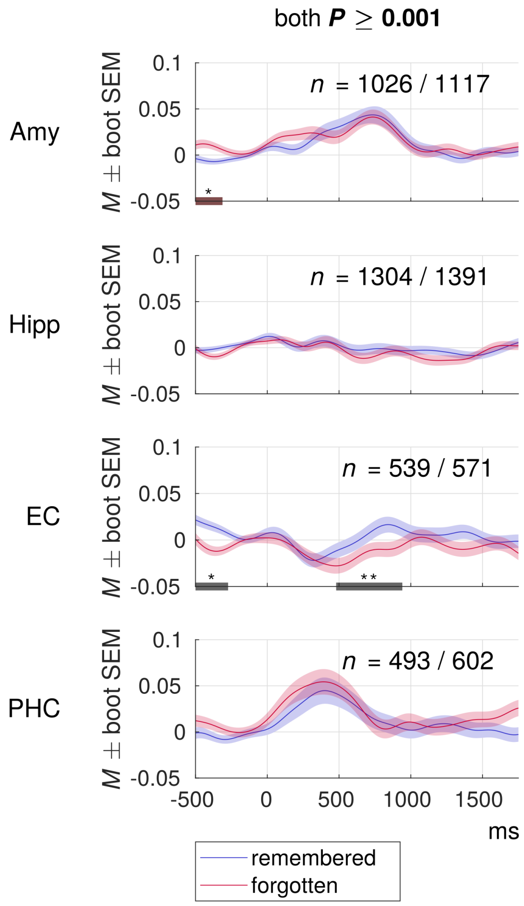

Figure S3. Unresponsive units with adjusted y axis range. Same data as shown in Figure 3 (right column).
Source data are provided in a git repository (see Data Availablity).

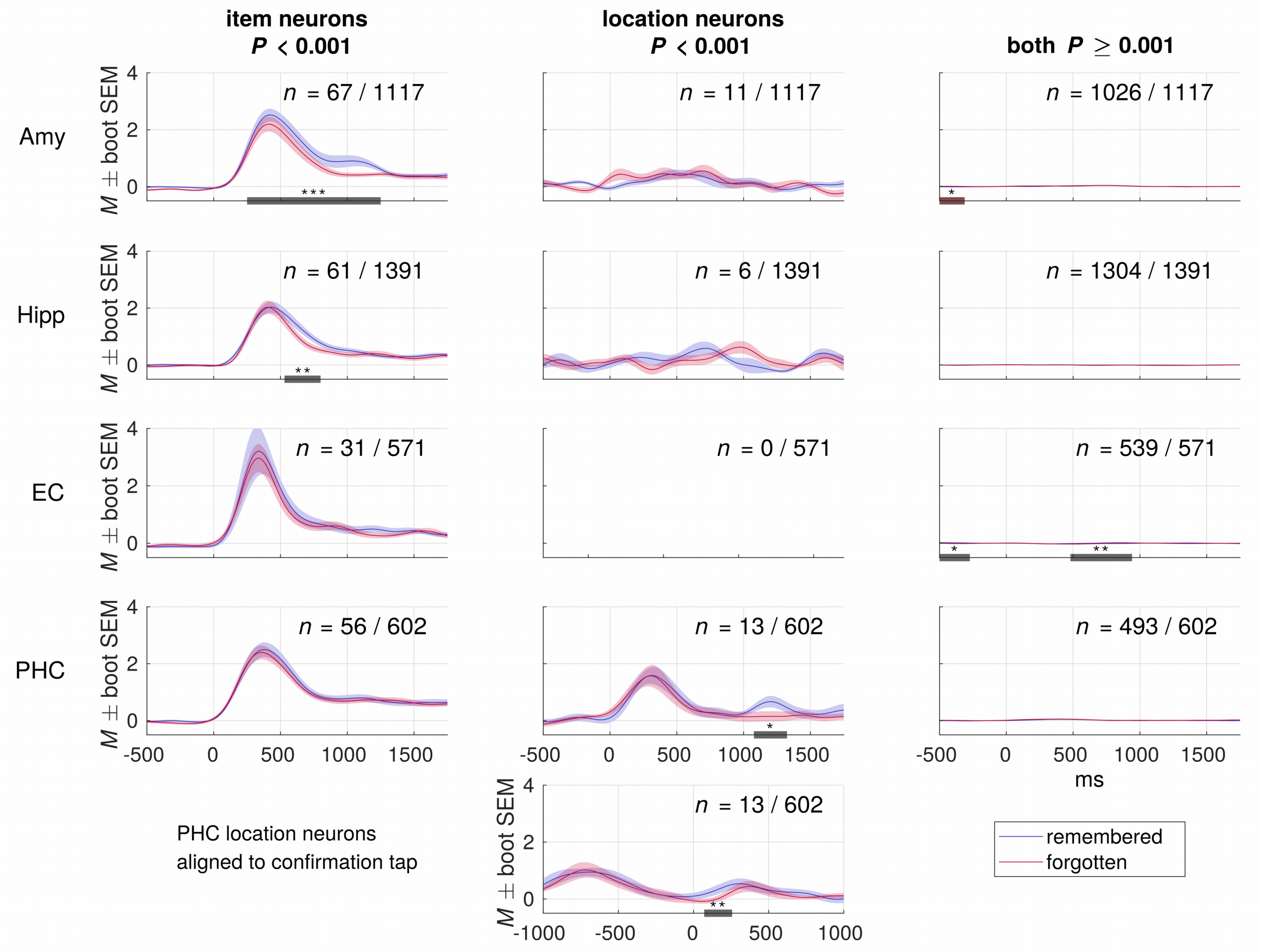

Figure S4. Excluding units with both response types. These are the same responses during encoding as in Figure 3. The inclusion criteria were a significant binwise ranksum test ($P < .001$) and responses to either items or locations, but not both.
Source data are provided in a git repository (see Data Availablity).

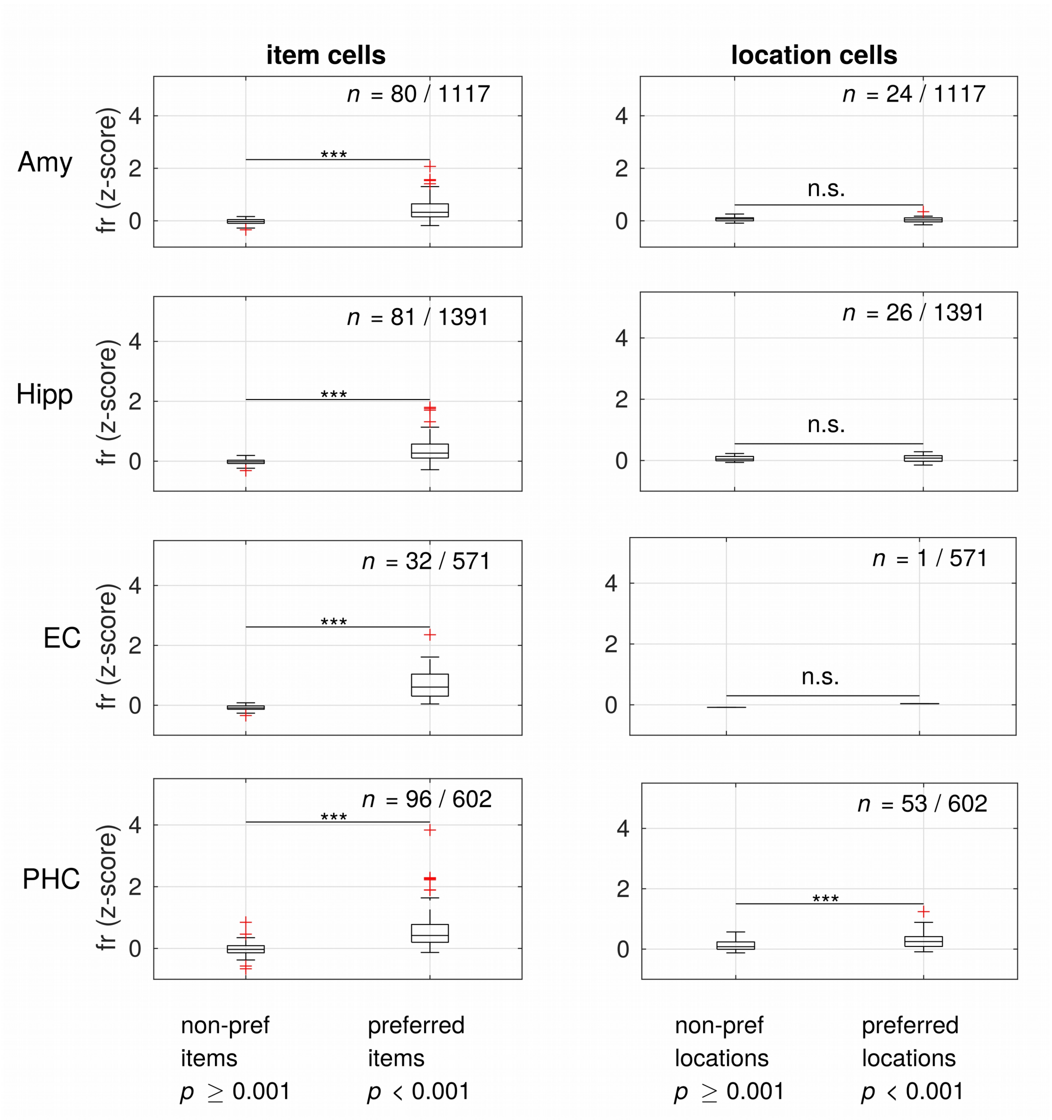

Figure S5. Firing rates during retrieval. Each box plot represents cells responding to items (left panels) or locations (right panels) during encoding trials. This figure shows their average firing rates (z-scores, normalized to a baseline period of 500 ms leading up to grid onset) during retrieval in a 1 second window leading up to the response tap. Two values are computed per cell, one for response-eliciting items or locations, one for non-response-eliciting items or locations (where ‚response-eliciting' refers to a significant response during encoding). The mean firing rates were compared using two-tailed signed-rank tests. *** $P < .001$, ** $P < .01$, * $P < .05$.
Source data are provided in a git repository (see Data Availablity).

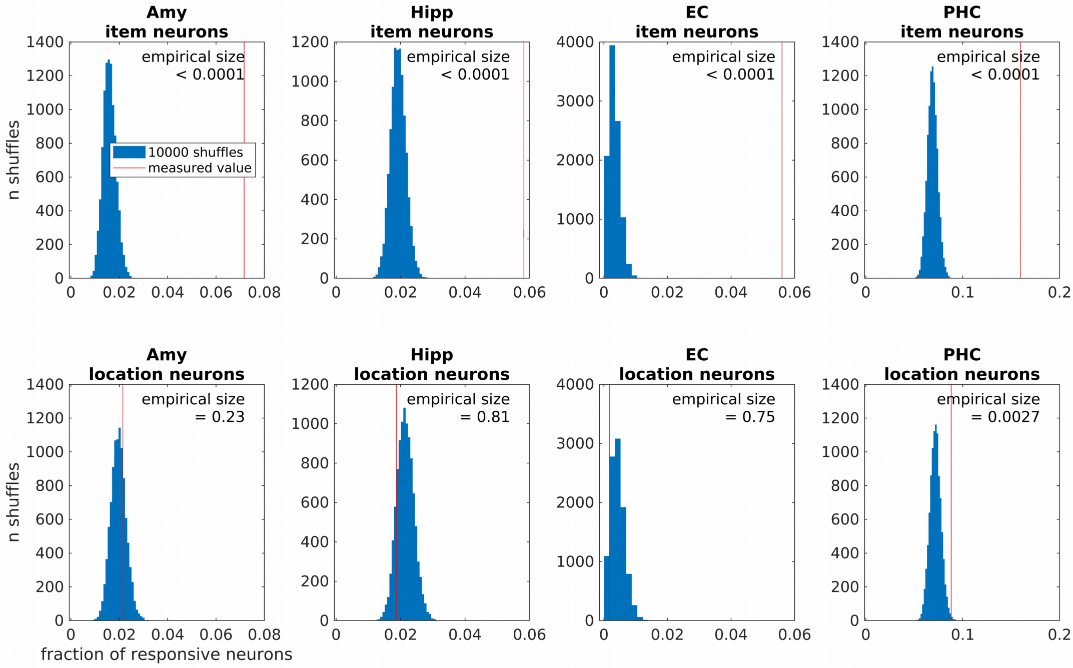

Figure S6. Empirical sizes of the permutation tests. The histograms show fractions of neurons producing item (top) or location (bottom) responses after label shuffling. Blue bars represent distributions of 10000 iterations, red lines represent the measured data.
Source data are provided in a git repository (see Data Availablity).

Patient	Session #	total units (single units)	n item cells Amy	n item cells Hipp	n item cells EC	n location cells PHC
1	1	121 (70)	0	5	4	0
1	2	120 (67)	1	1	0	1
1	3	57 (35)	0	1	0	0
2	1	69 (44)	2	2	0	0
2	2	65 (38)	4	1	0	0
2	3	47 (31)	2	0	0	0
2	4	32 (19)	1	0	0	0
2	5	38 (18)	0	1	0	0
2	6	46 (8)	0	0	0	0
3	1	71 (50)	0	1	0	0
3	2	83 (40)	5	3	0	0
3	3	88 (51)	3	4	0	0
3	4	56 (36)	3	5	0	0
3	5	64 (46)	4	2	0	0
4	1	86 (49)	1	1	0	0
4	2	67 (32)	0	0	1	0
4	3	26 (12)	0	0	0	0
5	1	115 (64)	3	2	0	3
5	2	108 (20)	1	2	0	3
6	1	105 (53)	3	0	2	0
6	2	102 (43)	6	1	4	0
6	3	74 (27)	0	0	1	0
6	4	66 (11)	0	1	1	0
6	5	32 (12)	0	0	0	0
7	1	154 (92)	0	5	7	5
7	2	95 (40)	1	2	2	3
7	3	90 (24)	0	1	0	1
7	4	74 (19)	0	0	4	2
7	5	79 (38)	0	4	2	4
8	1	75 (35)	3	0	0	0
8	2	76 (37)	2	2	0	0
8	3	47 (13)	4	1	0	0
8	4	31 (5)	3	1	0	0
9	1	64 (25)	2	0	0	1
10	1	152 (92)	0	2	0	1
10	2	131 (63)	1	5	0	0
10	3	101 (41)	0	3	1	2
11	1	152 (87)	3	0	0	6
11	2	133 (76)	2	5	0	2
11	3	110 (52)	1	2	0	8
12	1	97 (49)	8	1	1	0
12	2	114 (61)	6	6	2	4
12	3	85 (44)	4	5	0	1
13	1	83 (47)	1	3	0	6
	total	3681 (1816)	80	81	32	53

Table S1. Units recorded per session. Overview of how many units were recorded per session, and how many of them were single units (in parenthesis). The numbers of item and location cells in the different brain regions are also indicated.
